# Supplementary material for: Airway basal cells show a dedifferentiated KRT17highPhenotype and promote fibrosis in idiopathic pulmonary fibrosis
Source: Nat Commun. 2022 Sep 26;13:5637. doi: 10.1038/s41467-022-33193-0 (PMC9513076; doi:10.1038/s41467-022-33193-0)
Supplement: Supplementary file 1 — Supplementary Information [file 41467_2022_33193_MOESM1_ESM.pdf]

SUPPLEMENTARY INFORMATION

Airway Basal Cells show a dedifferentiated KRT17[high]Phenotype and promote Fibrosis in Idiopathic Pulmonary Fibrosis

Benedikt Jaeger, Ph.D.[1,2], Jonas Christian Schupp, M.D.[2,3,4], Linda Plappert[1,2], Oliver Terwolbeck[1,2], Nataliia Artysh [1], Gian Kayser, M.D.[5], Peggy Engelhard, Ph.D.[6], Taylor Sterling Adams, B.S.[3], Robert Zweigerdt, Ph.D.[7], Henning Kempf, Ph.D.[7], Stefan Lienenklaus, Ph.D.[8], Wiebke Garrels, Ph.D.[8], Irina Nazarenko, Ph.D.[9,10], Danny Jonigk, M.D.[2,11], Malgorzata Wygrecka, Ph.D[12], Denise Klatt, Ph.D.[13], Axel Schambach, M.D. Ph.D.[13,14], Naftali Kaminski, M.D.[3], Antje Prasse, M.D.[1,2,4]

[1] Fraunhofer Institute for Toxicology and Experimental Medicine, Hannover, Germany
[2] German Center for Lung Research, BREATH, Hannover, Germany
[3] Section of Pulmonary, Critical Care and Sleep Medicine, Yale School of Medicine, USA
[4] Department of Respiratory Medicine, Hannover Medical School, Hannover, Germany
[5] Institute of Surgical Pathology, University Medical Center, Freiburg, Germany
[6] Department of Pneumology, University Medical Center Freiburg, Germany
[7] Leibniz Research Laboratories for Biotechnology and Artificial Organs, Medical School Hannover, Germany
[8] Institute for Laboratory Animal Science, Hannover Medical School, Hannover, Germany.
[9] Institute for Infection Prevention and Hospital Epidemiology; Medical Center - University of Freiburg, Freiburg, Germany
[10] German Cancer Consortium (DKTK), Partner Site Freiburg and Cancer Research Center (DKFZ), Heidelberg, Germany
[11] Institute of Pathology, Medical School Hannover, Germany
[12] Department of Biochemistry, Faculty of Medicine, Justus Liebig University, Gießen, Germany
[13] Institute of Experimental Hematology, Hannover Medical School, Hannover, Germany
[14] Division of Hematology/Oncology, Boston Children's Hospital, Harvard Medical School, Boston, Massachusetts, USA

Supplementary Tables

Supplementary Table 1. Baseline Characteristics of IPF Patients and nonUIP ILD patients of the single cell sequencing cohort

Supplementary Table 1. Baseline Characteristics of IPF and nonUIP ILD*

Characteristic	IPF N=9	NonUIP ILD N=6
Age -yr	71 ± 7	64 ± 11
Male/ female sex	7/2	6/0
FVC percent predicted value	71 ± 9	69 ± 27
DLCO percent predicted	54 ± 13	53 ± 20
Smoking Status		
Never smoked -%	3	3
Former smoker-%	6	3
Current smoker-%	0	0

* Plus–minus values are means ±SD. FVC denotes for forced vital capacity. DLCO denotes for diffusion capacity. NonUIP ILD denotes for patients with a fibrotic interstitial lung disease (ILD) who presented with a computer tomography (CT) scan that is not consistent with a definite Usual Interstitial Pneumonia (UIP) pattern. IPF denotes for idiopathic pulmonary fibrosis.

Supplementary Table 2: Primary antibodies used for flow cytometry

Marker	Vendor	Dilution/ Conc.
Epcam APC	Miltenyi,<br># 130-11-000	1:50
Cytokeratin 5 PE	abcam<br># ab224985	1:50
Cytokeratin 17 A488	abcam,<br># ab185032	1:50
PROM1 APC	Miltenyi, # 130-113-668	1:100

Supplementary Table 3: Primary antibodies used for IHC

Marker	Clone	Vendor	Procedure	Dilution/ Conc.	Incubation	Detection
C-src	polyclonal anti-src rabbit antibody	Abcam, #ab47405	Manual	1:50	30 min, RT	AP, (DAKO REAL Streptavidin Alkaline Phosphatase)
CK5/6	monoclonal mouse anti-human CK5/6 (DAKO clone D5/16 B4)	Dako Omnis, #GA78061-2	Manual	Ready to use	30 min, RT	AP, (DAKO REAL Streptavidin Alkaline Phosphatase)
P63	P63 polyclonal rabbit anti-human antibody	Calbiochem, #PC373	Manual	1:1000	30 min, RT	HRP, Peroxidase coupled secondary antibody (DAKO EnVision FLEX/HRP)
anti-human anti-αvβ6	MAb6.3G9, primary monoclonal rat antibody	kindley provided by Shelia Violette Biogen Idec	Manual	1:100	30 min, RT	HRP, Peroxidase coupled secondary antibody (DAKO EnVision FLEX/HRP)
negative control	Negative Control Mouse IgG1 antibody	Dako Omnis, #GA75066-2	Manual	1:20	overnight,4°C	AP, (DAKO REAL Streptavidin Alkaline Phosphatase)
negative control	FLEX Universal Negative Control, Rabbit	Dako Omnis, #IS60061-2	Manual	1:20	overnight,4°C	AP, (DAKO REAL Streptavidin Alkaline Phosphatase)
negative control	Negative Control Mouse IgG1 antibody	Dako Omnis, #GA75066-2	Manual	1:20	overnight,4°C	AP, (DAKO REAL Streptavidin Alkaline Phosphatase)

Supplementary Table 4: Secondary antibodies used for IHC

Clone	Vendor	Procedure	Dilution/ Conc.	Incubation
Goat IgG anti-Mouse IgG (H+L)-Biotin	Dianova, #115-065-003	Manual	1:800	30 min RT
Goat IgG anti-Rabbit IgG (H+L)-Biotin	Dianova, #111-065-003	Manual	1:800	30 min RT

Supplementary Table 5: Primary and secondary antibodies used for immunofluorescence staining

Sections	Marker	Clone	Vendor	Dilution	Detection Ab	Vendor
Human lung tissue	c-src	Src (36D10) Rabbit mAb	Cell Signaling #2109S	1:400	Donkey IgG anti-Rabbit IgG (H+L)-Cy5	Dianova, #711-175-152
Human lung tissue	CK5/6	FLEX Monoclonal Mouse Anti-Human Cytokeratin 5/6 Clone D5/16 B4	Dako Omnis, #GA78061-2	Ready-to-Use	Donkey Fab anti-Mouse IgG (H+L)-Alexa Fluor 488	Dianova, #715-547-003
NSG-Venus Mice lung tissue	CK8	Rabbit Anti-Cytokeratin 8 antibody [EP1628Y]	Abcam, #ab53280	1:100	Donkey IgG anti-Rabbit IgG (H+L)-Cy3	Dianova, #711-165-152
NSG-Venus Mice lung tissue	CK5/6	FLEX Monoclonal Mouse Anti-Human Cytokeratin 5/6 Clone D5/16 B4	Dako Omnis, #GA78061-2	Ready-to-Use	Donkey IgG anti-Mouse IgG (H+L)-Cy5	Dianova, #715-175-150
NRG mice lung tissue	AQP5	Goat AQP5 Antibody (G-19)	Santa Cruz, #sc-9891	1:25	Donkey IgG anti-Goat IgG (H+L)-Cy3	Dianova, #705-165-147
NRG mice lung tissue	eGFP	GFP (D5.1) XP® Rabbit mAb	Cell Signalling, #2956	1:75	Donkey Fab anti-Rabbit IgG (H+L)-Alexa Fluor 488	Dianova, #711-547-003
NRG mice lung tissue	CK5/6	FLEX Monoclonal Mouse Anti-Human Cytokeratin 5/6 Clone D5/16 B4	Dako Omnis, #GA78061-2	Ready-to-Use	Donkey Fab anti-Mouse IgG (H+L)-Alexa Fluor 488	Dianova, #715-547-003
Cryo Slide/ 3D Organoids w/o IPF fibroblasts	CK5/6	FLEX Monoclonal Mouse anti-human cytokeratin 5/6 Clone D5/16 B4	Dako Omnis, #GA78061-2	Ready-to-Use	Alexa Fluor® 488-conjugated AffiniPure Fab fragment donkey anti mouse IgG	Dianova, #715-547-003

Sections	Marker	Clone	Vendor	Dilution	Detection Ab	Vendor
Cryo Slide/ 3D Organoids w/o IPF fibroblasts	Anti-alpha smooth muscle Actin	Rabbit monoclonal	Abcam,# ab124964	1:400	Cy™5 AffiniPure DonkeyAnti-RabbitIgG (H+L)	Jackson immuno research, #711-175-152
Cryo Slide/ 3D Organoids w/o IPF fibroblasts	Vimentin	goat	Merck, #V4630	1:20	Donkey IgG anti-Goat IgG (H+L)-Cy3	Dianova, #705-165-147
	Isotype control	Rabbit IgG, polyclonal – Isotype Control	Abcam, #ab37415	1:400	Cy™5 AffiniPure DonkeyAnti-RabbitIgG (H+L)	Jackson immuno research, #711-175-152
	Negative control	FLEX Universal Negative Control, Mouse	Dako Omnis, #GA75066-2	Ready-to-Use	Donkey Fab anti-Mouse IgG (H+L)-Alexa Fluor 488	Dianova, #715-547-003
	Isotype Control	Goat IgG Isotype Control	Thermo Fisher Scientific, #31245	1:25	Donkey IgG anti-Goat IgG (H+L)-Cy3	Dianova, #705-165-147
Cryo Slide/ 3D Organoids w/o IPF fibroblasts	CK5	Alexa Fluor® 647 Rabbit monoclonal [EP1601Y] to Cytokeratin 5	Abcam, #ab 193895	1:200	-	-
Cryo Slide/ 3D Organoids w/o IPF fibroblasts	CK6	Rabbit monoclonal [EPR1602Y] to Cytokeratin 6	Abcam, # ab93279	1:50	Cy™3 AffiniPure Donkey Anti-Rabbit IgG (H+L)	Jackson immuno research, #711-165-152
Cryo Slide/ 3D Organoids w/o IPF fibroblasts	MUC5AC	Rabbit Polyclonal Antibody, Cy3 Conjugated	Bioss Antibodies, # bs-7166R-Cy3	1:50	-	-
Cryo Slide/ 3D Organoids w/o IPF fibroblasts	Acetyl-α-Tubulin (Lys40)	Mouse monoclonal antibody	Cell signaling, #12152	1:500	Cy™5 AffiniPure Donkey Anti-Mouse IgG (H+L)	Jackson immuno research, #715-175-150
Cryo Slide/ 3D Organoids w/o IPF fibroblasts	CK17	Mouse monoclonal antibody	NSJ Bioreagent s	1:200	Cy™3 AffiniPure DonkeyAnti-Mouse IgG (H+L)	Jackson immuno research, #715-165-150

Supplementary Table 6: Primary antibodies used for Western Blot

Marker	Clone	Vendor	Dilution/ Conc.
c-src	polyclonal anti-src rabbit antibody	Abcam, #ab47405	1:500
Phospho-Src	Phospho-Src (Tyr527) Antibody	Cell Signaling #2105S	1:1000
Type I Collagen	polyclonal goat anti-Type I Collagen	Southern Biotec, #SAB-1310-01	1:1000
α-SMA	monoclonal mouse anti-α-SMA, ASM-1	Merck Millipore, #CBL171	1:2000
Fibronectin,cFn	monoclonal mouse anti-FN, DH1	Enzo Life Sciences, #BML-FG6010	1:250
p44/42 (Thr202/Tyr204)	mouse anti-phospho-p44/42 (Thr202/Tyr204)	Cell Signaling, #9106	1:500
total p44/42	rabbit mAb anti total p44/42	Cell Signaling, #4695	1:1000
phospho-EGFR (Tyr845)	rabbit phospho-EGFR (Tyr845)	Zytomed, #205-0235	1:500
total EGFR	rabbit anti-total EGFR	Biomol	1:500
GAPDH (6C5)	mouse monoclonal antibody raised against GAPDH	Santa Cruz, #sc-32233	1:200
α-Tubulin	Rabbit anti- α-tubulin antibody	Cell Signaling, #2125	1:1000

Supplementary Table 7: Secondary antibodies used for Western Blot

Clone	Vendor	Dilution/ Conc.
goat anti rabbit (H+L)-HRP conjugate	BioRad, #1706515	1:3000
goat anti mouse (H+L)-HRP conjugate	BioRad, #1706516	1:3000

Supplementary Figures

Supplementary Figure 1

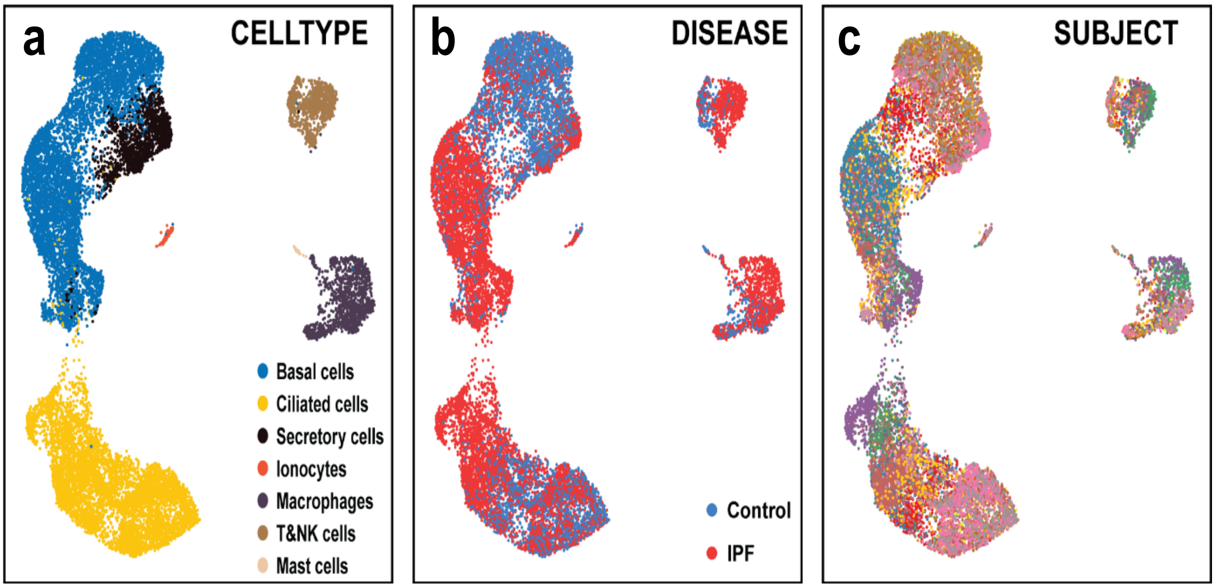

Supplementary Fig. 1. *Uniform Manifold Approximation and Projection (UMAP) of the full dataset of 17,339 single cell transcriptomes visualizes the seven major discrete cell types detected.* ***a,*** *UMAPs colored by cell types.* ***b,*** *UMAPs colored by disease states.* ***c,*** *UMAPs colored by subjects.*

Supplementary Figure 2

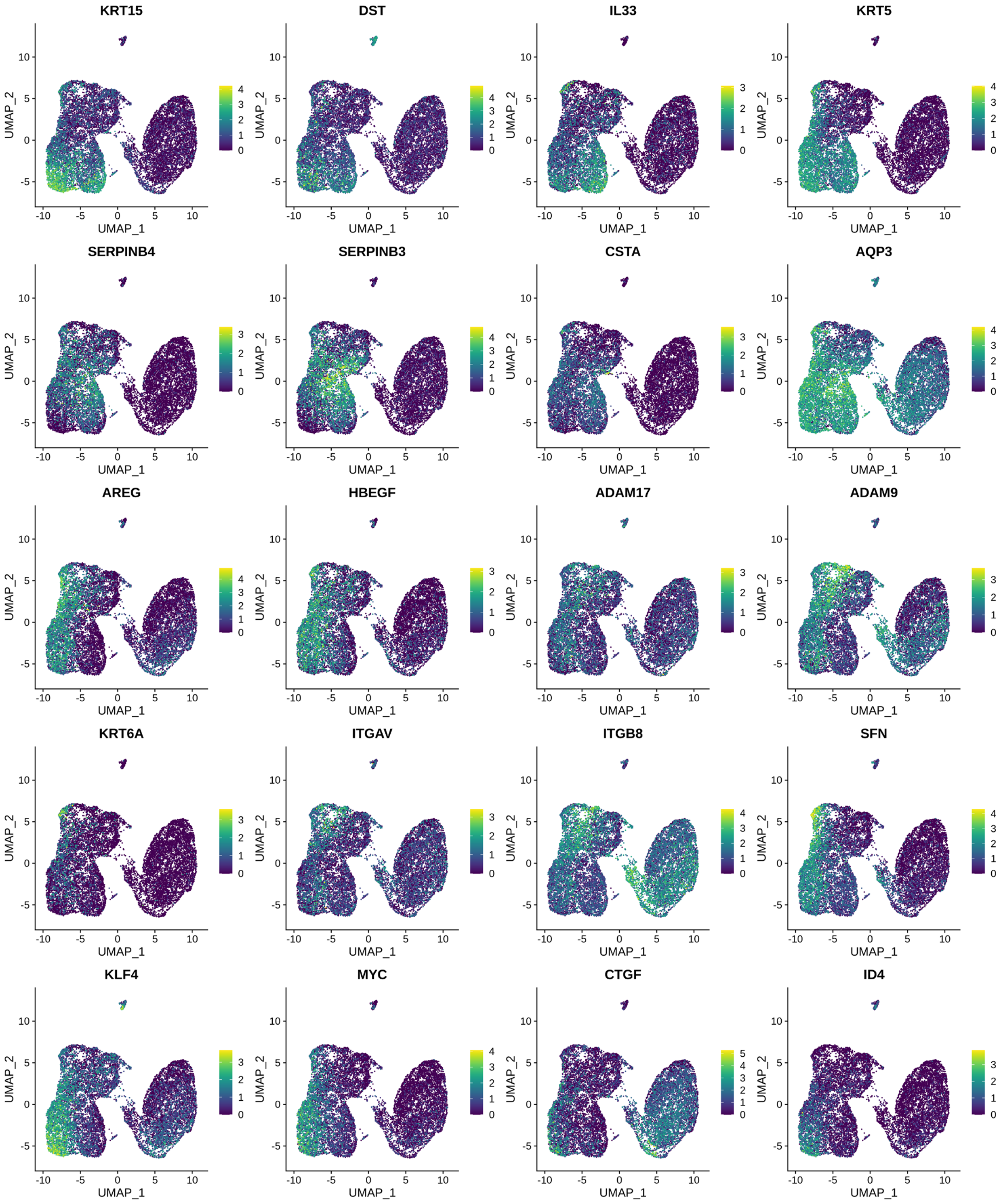

Supplementary Fig. 2: *Expression of selected key genes of (IPF-)ABCs. UMAPs of epithelial cells colored by gene expression values of features that are associated with basal cells or with increased expression in IPF-ABCs compared to controls.*

Supplementary Figure 3

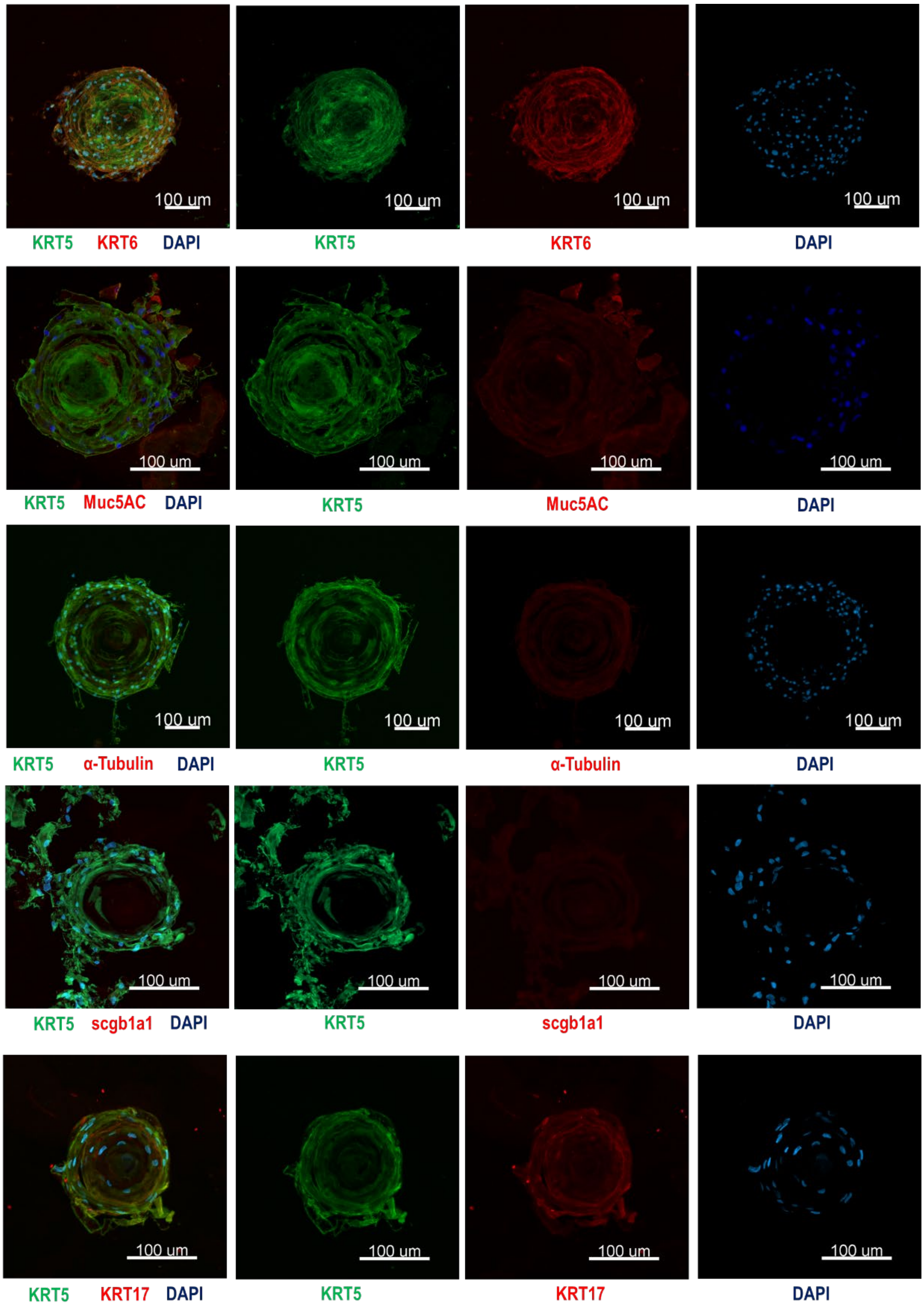

Supplementary Fig. 3. Cellular composition of bronchospheres generated by IPF-ABCs. IPF-ABCs were cultured in matrigel applying a transwell system for 21 days as described**.** *Overlay and single original registrations of the generated bronchospheres obtained by confocal laser microscopy are depicted. At day 21 the bronchospheres consisted mainly out of KRT5+KRT6+KRT17+ basal cells. Only few cells expressed Muc5AC, a marker for secretory cells. Using our conditions, we did not observe differentitation into a-tubulin positive ciliated cells or scgb1A1 positive club cells. Representative registrations of five independent experiments are shown.*

Supplementary Figure 4

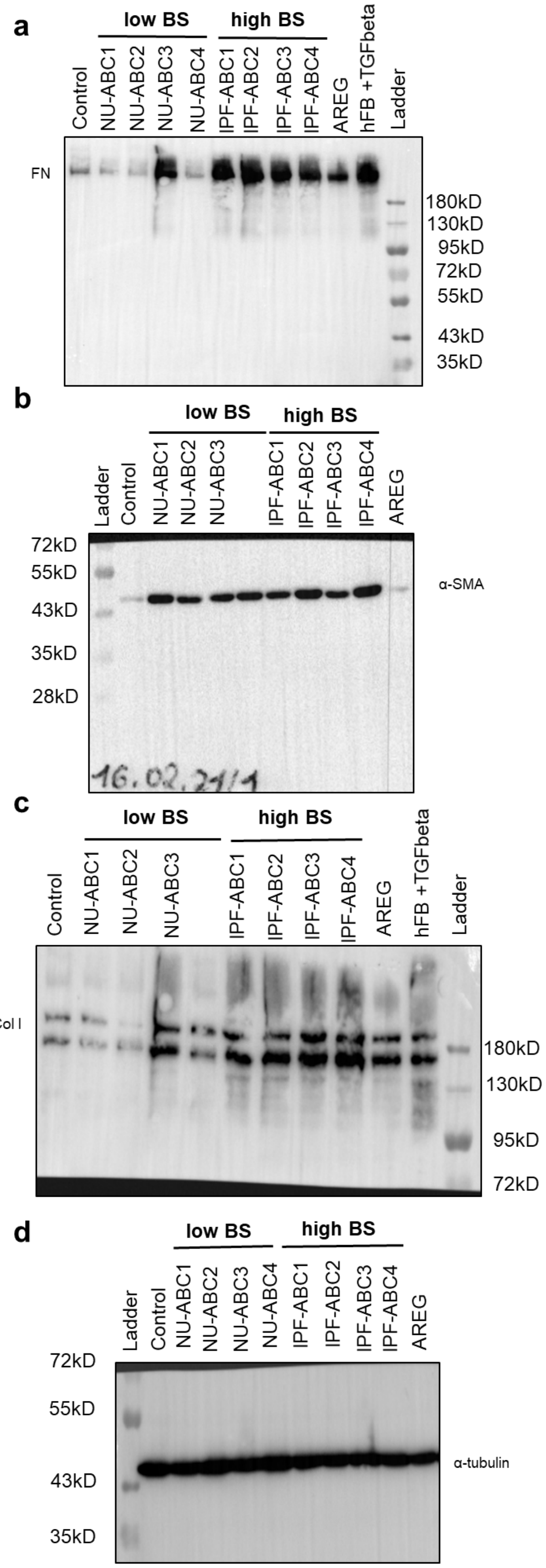

Supplementary Fig. 4. *Uncropped version of the blots shown in* ***Fig. 2m*** *of the manuscript. Uncropped blot depicting* ***a,*** *fibronectin,* ***b,*** *α-SMA and* ***c,*** *collagen I and* ***d,*** *α-tubulin expression of lung fibroblasts, which were stimulated with conditioned media obtained from NU-ABC (low bronchosphere counts) or IPF-ABC (high bronchosphere counts) harvested at day 14.*

Supplementary Figure 5

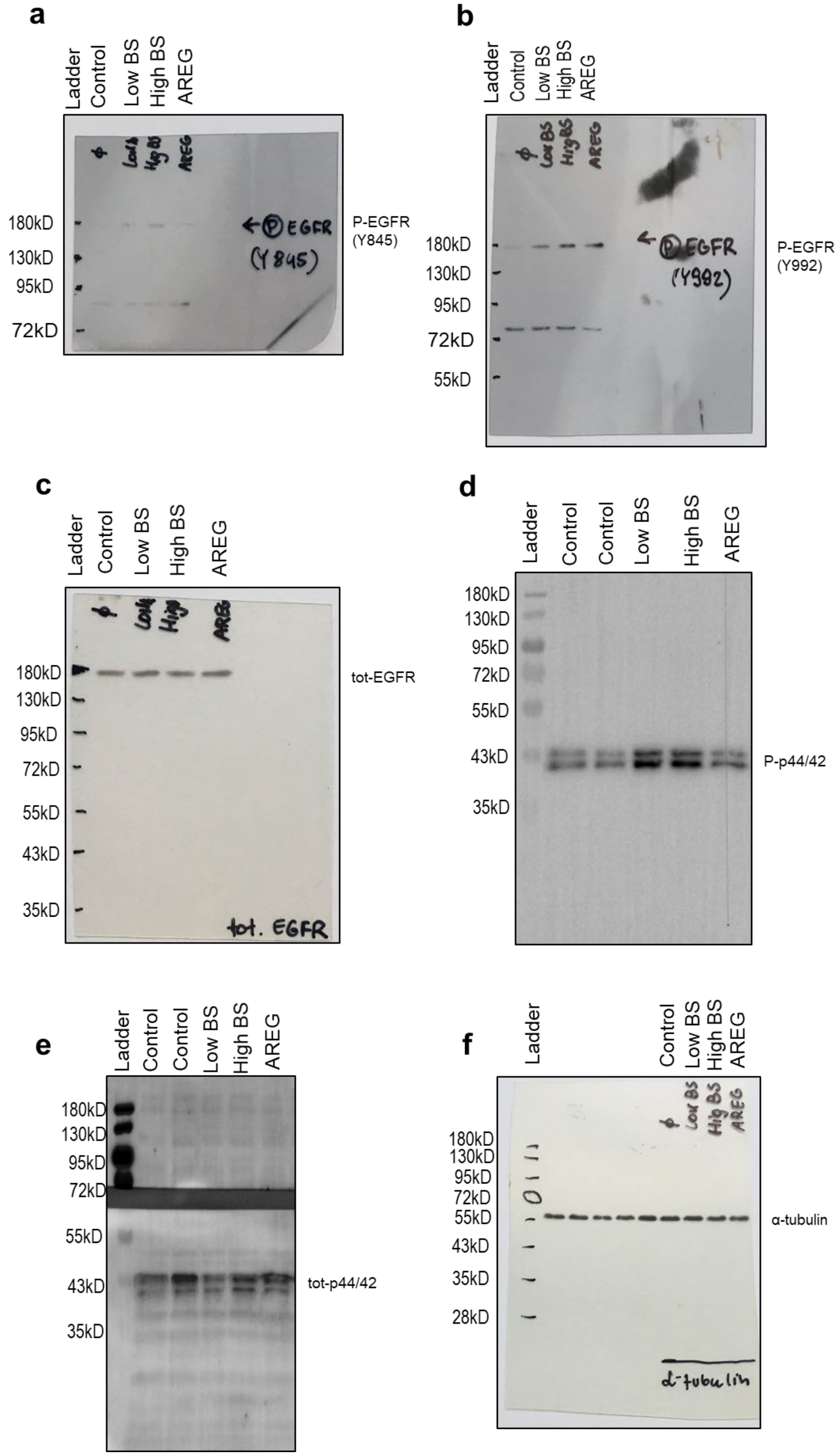

Supplementary Fig. 5. *Uncropped version of the blot shown in* ***Fig. 2p*** *of the manuscript.* ***a,b,c,d,e*** *EGFR phosphorylation and* ***f,*** *α-tubulin expression in lung fibroblasts which were cultured in pooled conditioned media of bronchospheres derived from IPF-ABCs (High BS) in comparison to conditioned media of bronchospheres derived from NU-ABCs (Low BS).*

Supplementary Figure 6

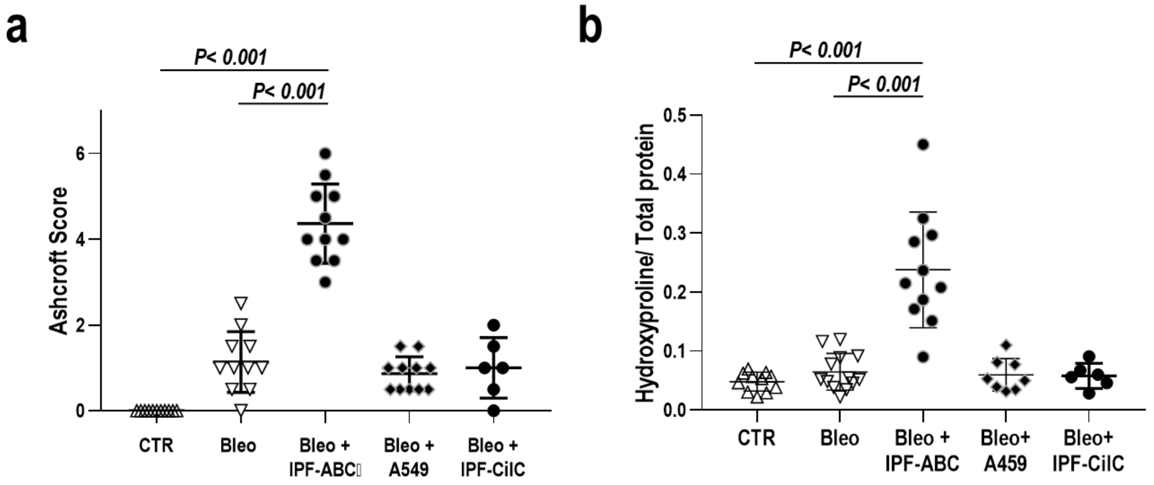

Supplementary Fig. 6. Airway basal cells of IPF patients (IPF-ABC) but not A549 or ciliated cells augment fibrosis in bleomycin challenged NRG mice. *NRG mice received either PBS (CTR) or bleomycin (Bleo) intratracheally and some mice (Bleo + IPF-ABC) additionally three days later 200.000 IPF-ABCs, A549 or ciliated epithelial cells (CilC) intratracheally. Ciliated cells were isolated from air liquid interface (ALI) cultures derived from IPF-ABCs. Lungs of mice were harvested for either histopathological scoring (Ashcroft (mean ± SD),* ***a,*** *or hydroxyproline measurements (mean ± SD)* ***b,*** *CTR denotes for control mice; Bleo denotes for bleomycin; IPF-ABC denotes for airway basal cells derived from IPF patients. IPF-CilC denotes for ciliated epithelial cells derived from IPF patients. One-way ANOVA with Tukey correction for multiple testing (a,b).*

Supplementary Figure 7

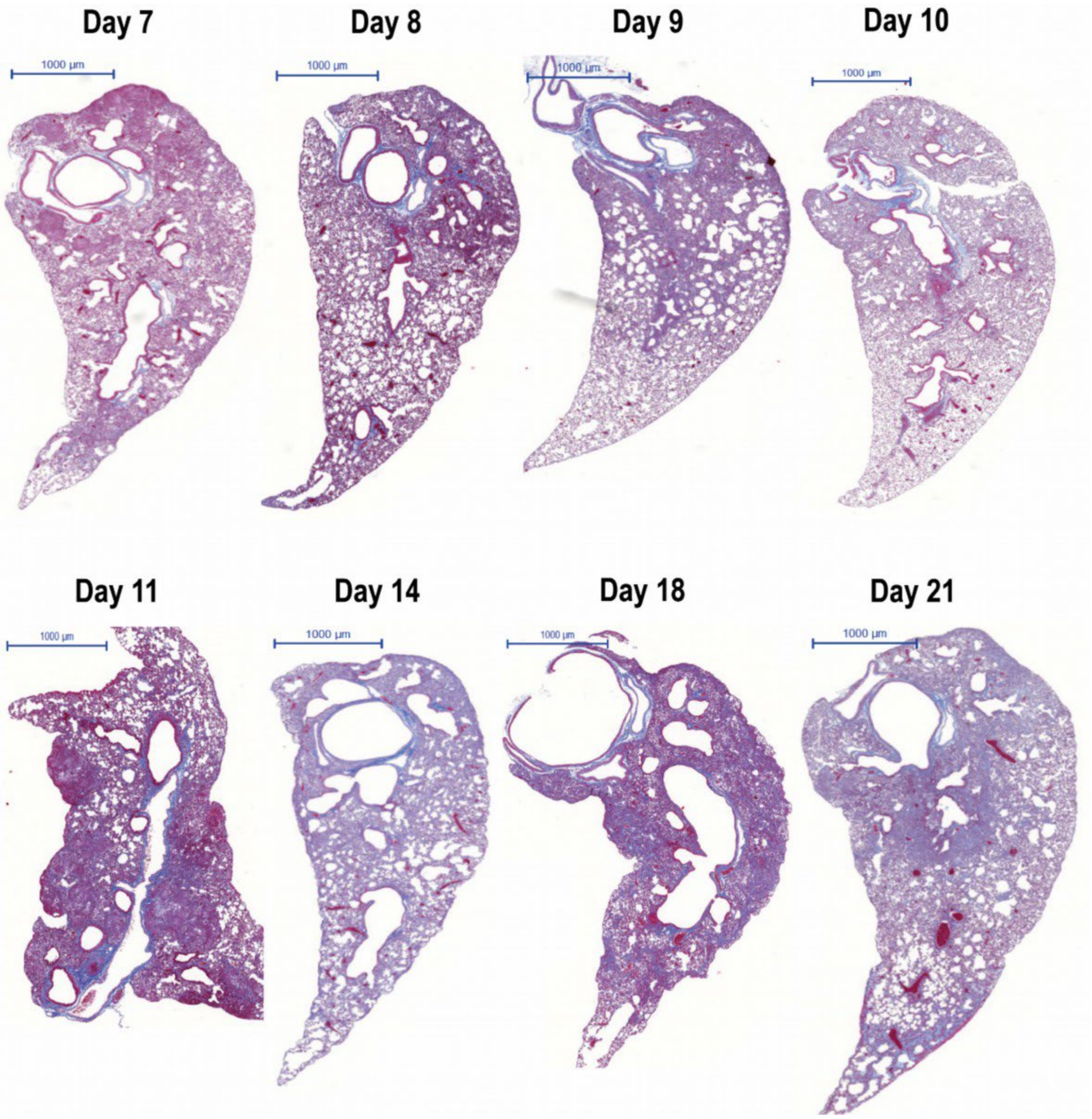

Supplementary Fig. 7: Severely affected mice had to be euthanized at different time points and lungs of these mice suggest that fibrotic remodeling is visible from day 8. *NRG mice received bleomycin per intratracheal (i.t.) injection and human IPF-ABCs derived from different patients were administered i.t. three days later. There is heterogeneity in regards to the induced fibrotic remodeling, evolution of cystic lesions and traction bronchiectasis as well as evolution of dysplastic epithelial lesions between the human IPF-ABC lines. No systematic experiment with no replicates. Shown are lungs from mice that had to be euthanized prior to day 21.*

Supplementary Figure 8

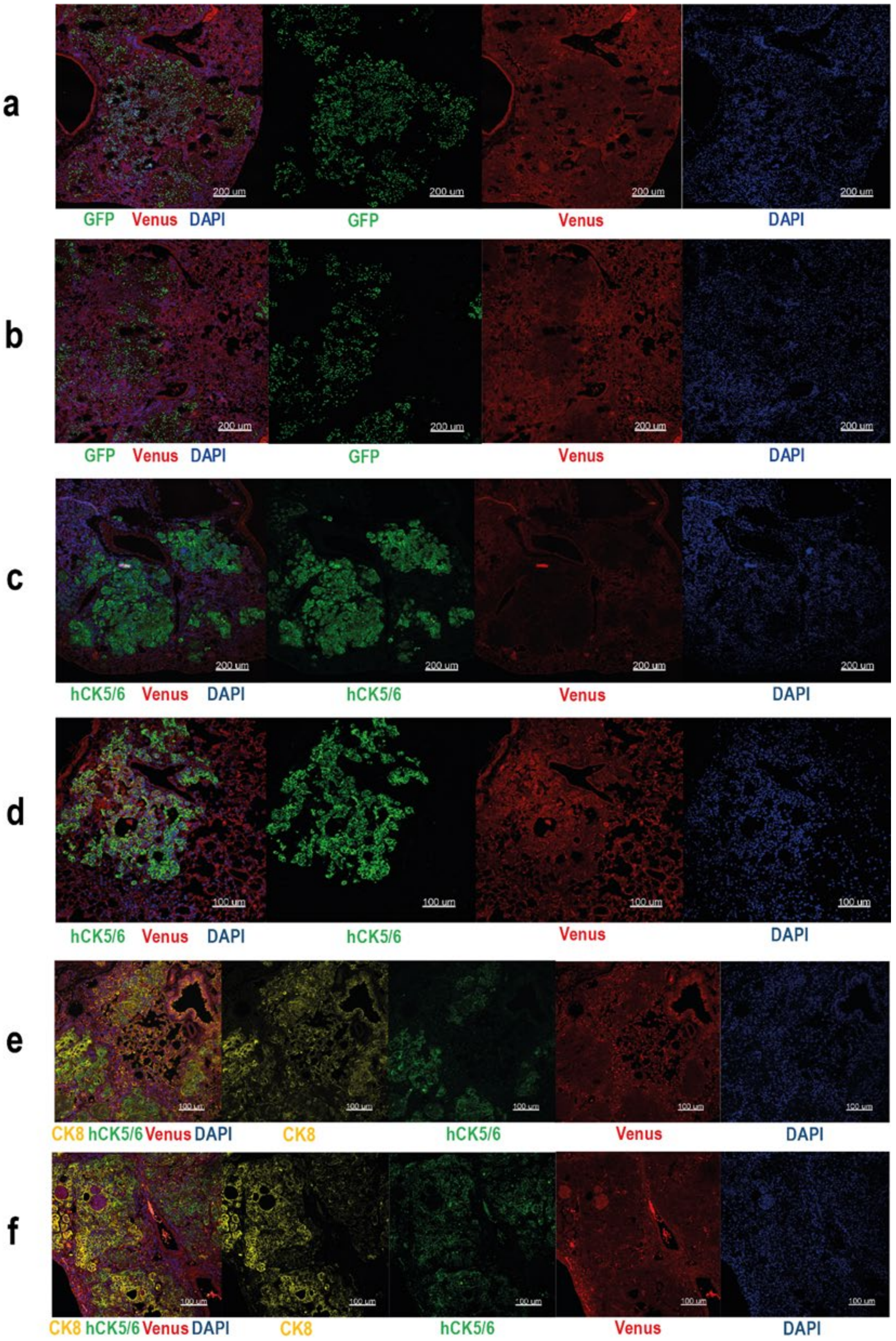

Supplementary Fig. 8. Overlay and single original registrations obtained by confocal laser microscopy which are depicted in Fig. 2i. *NSG mice received bleomycin i.t. at day 0 and three days later human IPF-ABCs which were transduced with a lentiviral vector encoding for eGFP. Lungs were harvested at day 21.* ***a,b,*** *Shown are eGFP staining in green, Venus expression in red, and nuclei staining by DAPI in blue.* ***c,d,*** *Shown are human cytokeratin 5/6 (hCK5/6) staining in green, Venus expression in red and nuclei staining by DAPI in blue* ***e,f,*** *Shown are human cytokeratin 5/6 (hCK5/6) staining in green, cytokeratin 8 (CK8) staining in yellow, Venus expression in red and nuclei staining by DAPI in blue. Scale bars, 50μm. Representative registrations of six independent experiments with similar results are shown.*

Supplementary Figure 9

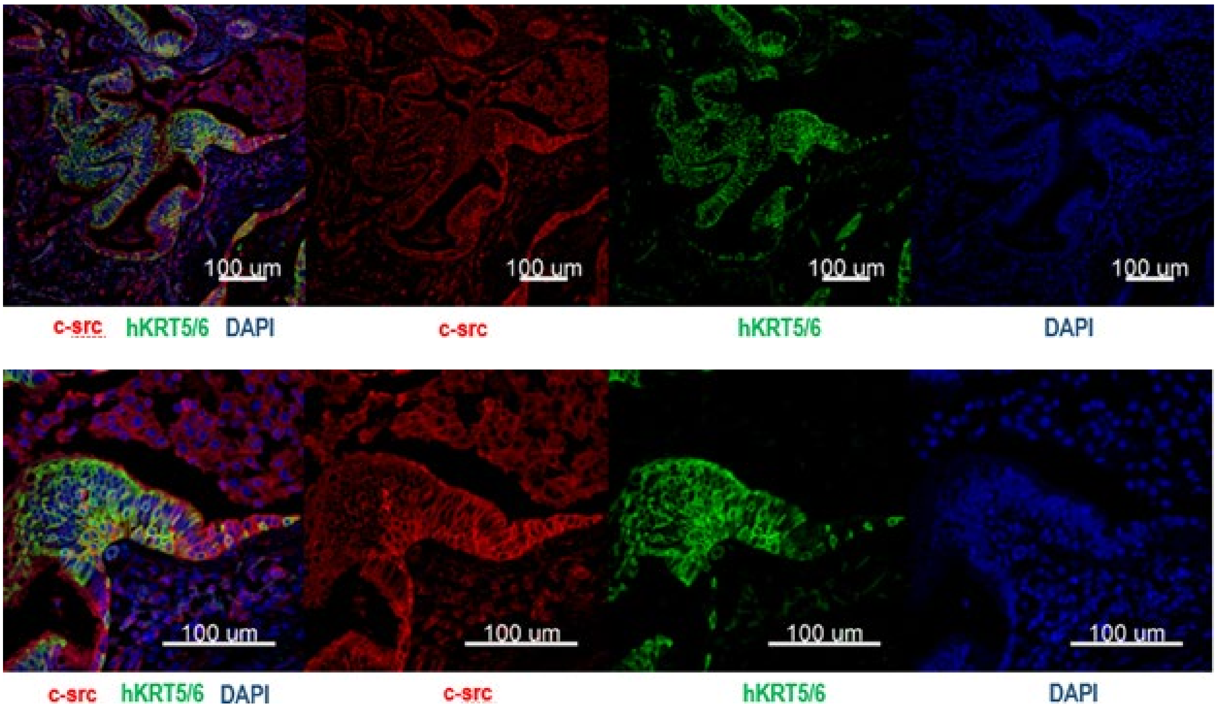

Supplementary Fig. 9. C-SRC is highly expressed in human lung tissues derived from patients with IPF. *Overlay and single original registrations of human IPF lung tissue obtained by confocal laser microscopy which are depicted in Fig. 4B. Additional original registrations with higher magnification are depicted in the lower panel. KRT5/6 staining signal in green, c-src staining in red and nuclei staining by DAPI in blue. Representative registrations of ten independent experiments with similar results are shown.*

Supplementary Figure 10

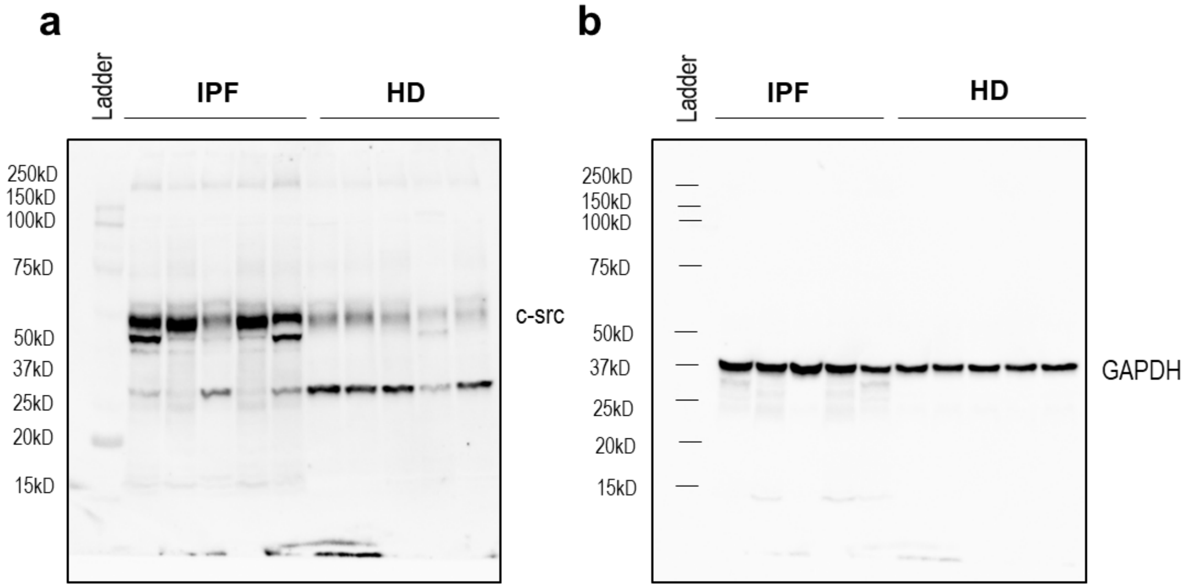

Supplementary Fig. 10. *Uncropped version of the cropped blot shown in* ***Fig. 4d****. of the manuscript.* ***a,*** *un-cropped blot depicting c-src protein expression and* ***b,*** *uncropped blot detecting GAPDH expression in lung tissue homogenates of IPF-patients compared to healthy donors.*

Supplementary Figure 11

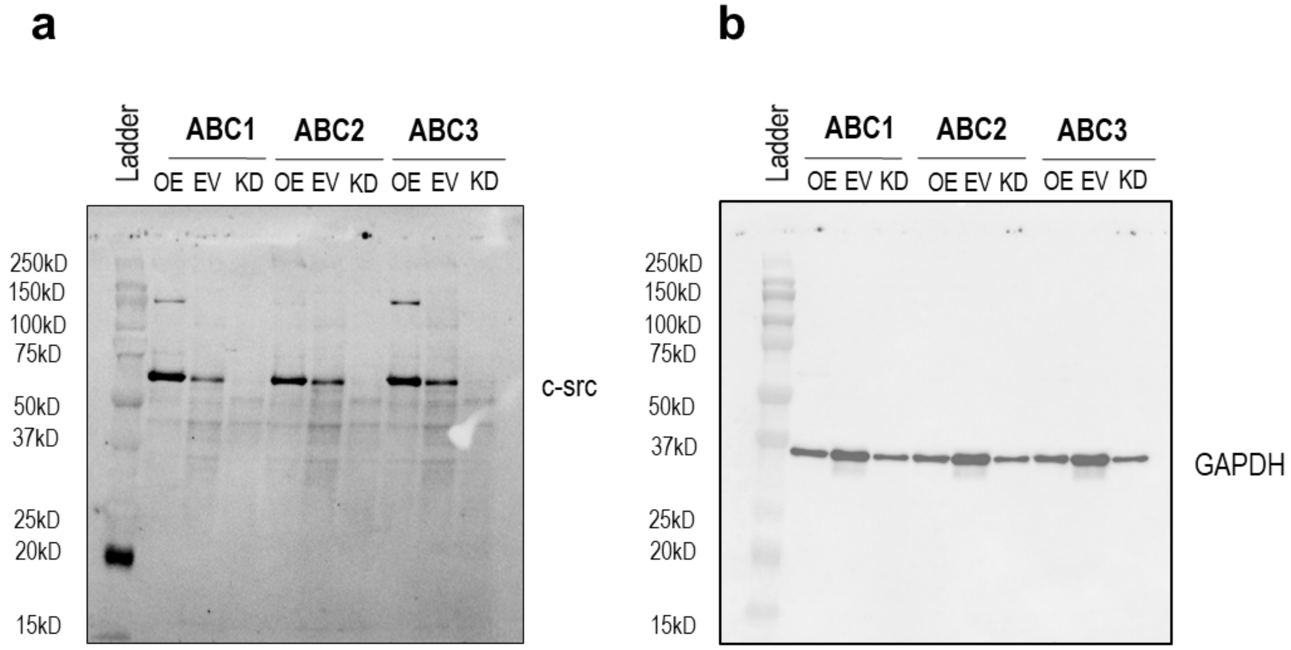

Supplementary Fig. 11. *Uncropped version of the cropped blot shown in **Fig. 4f** of the manuscript. **a,** uncropped blot of c-src protein expression and **b,** uncropped blot of GAPDH expression in untouched, lentiviral vector transduced or knockdown of human IPF-ABCs.*

Supplementary Figure 12

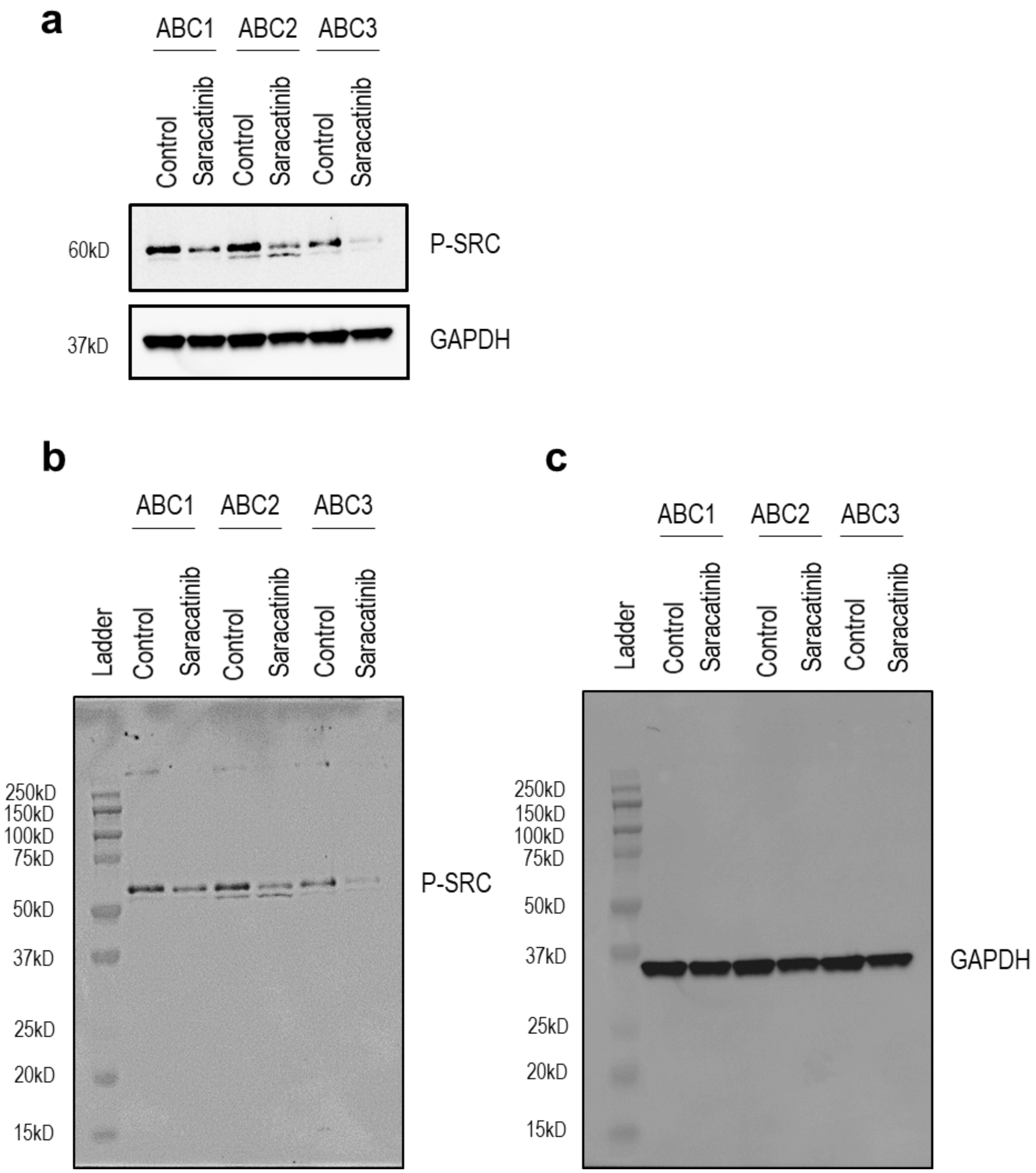

Supplementary Fig. 12. *Down-regulation of phosphorylated c-src by saracatinib treatment in IPF-ABCs. Depicted are cropped and uncropped blots of IPF-ABCs which were either untreated or saracatinib treated.* ***a,*** *cropped version of the blot which shows down-regulation of phosphorylated c-src in IPF-ABCs by saracatinib treatment. A representative blot of two independent experiments with similar results is shown.* ***b,*** *uncropped version of the blot which depicts pospho-src.* ***c,*** *uncropped version of the blot which depicts GAPDH expression.*

Supplementary Figure 13

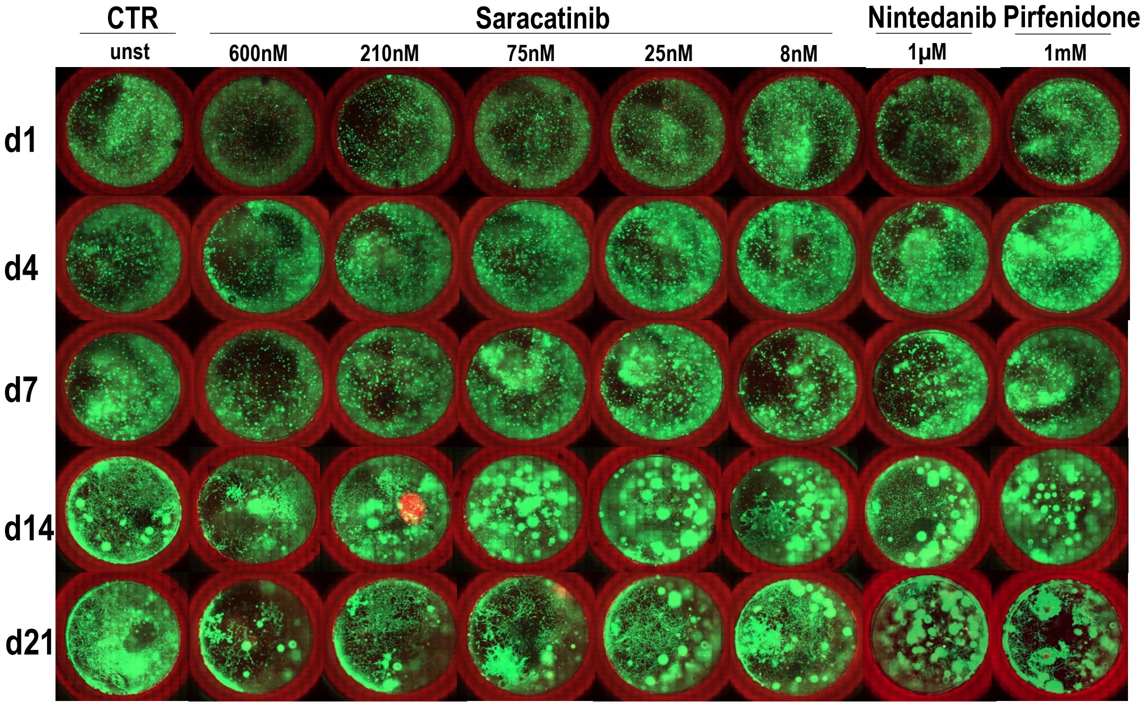

Supplementary Fig. 13. Live death staining of bronchosphere cultures with IPF lung fibroblasts. *Green staining detects vivid cells while red staining detects dead cells. In all bronchosphere cultures w/wo treatment with saracatinib, pirfenidone or nintedanib hardly any dead cells were detected at day 4 (d4), day 7 (d7), day 14 (d14), and day 21 (d21). Representative microphotographs of three independent experiments with similar results are depicted.*
