# Supplementary material for: Airway basal cells show a dedifferentiated KRT17highPhenotype and promote fibrosis in idiopathic pulmonary fibrosis
Source: Nat Commun. 2022 Sep 26;13:5637. doi: 10.1038/s41467-022-33193-0 (PMC9513076; doi:10.1038/s41467-022-33193-0)
Supplement: Supplementary file 2 — Reporting Summary [file 41467_2022_33193_MOESM2_ESM.pdf]

nature research

Corresponding author(s): Antje Prasse

Last updated by author(s): Aug 21, 2022

Reporting Summary

Nature Research wishes to improve the reproducibility of the work that we publish. This form provides structure for consistency and transparency in reporting. For further information on Nature Research policies, see our Editorial Policies and the Editorial Policy Checklist.

Statistics

For all statistical analyses, confirm that the following items are present in the figure legend, table legend, main text, or Methods section.

n/a	Confirmed	
☐	☒	The exact sample size (*n*) for each experimental group/condition, given as a discrete number and unit of measurement
☐	☒	A statement on whether measurements were taken from distinct samples or whether the same sample was measured repeatedly
☐	☒	The statistical test(s) used AND whether they are one- or two-sided<br>*Only common tests should be described solely by name; describe more complex techniques in the Methods section.*
☒	☐	A description of all covariates tested
☐	☒	A description of any assumptions or corrections, such as tests of normality and adjustment for multiple comparisons
☐	☒	A full description of the statistical parameters including central tendency (e.g. means) or other basic estimates (e.g. regression coefficient) AND variation (e.g. standard deviation) or associated estimates of uncertainty (e.g. confidence intervals)
☐	☒	For null hypothesis testing, the test statistic (e.g. *F*, *t*, *r*) with confidence intervals, effect sizes, degrees of freedom and *P* value noted<br>*Give P values as exact values whenever suitable.*
☒	☐	For Bayesian analysis, information on the choice of priors and Markov chain Monte Carlo settings
☐	☒	For hierarchical and complex designs, identification of the appropriate level for tests and full reporting of outcomes
☐	☒	Estimates of effect sizes (e.g. Cohen's *d*, Pearson's *r*), indicating how they were calculated

Our web collection on statistics for biologists contains articles on many of the points above.

Software and code

Policy information about availability of computer code

Data collection

For brightfield and fluorescence microscopy:
The ZEN 2.5 (blue edition) software (Zeiss/Germany) was used for brightfield image analyses and brightfield image evaluation.

Confocal laser microscopy images were analyzed and processed with software Imaris 7.6 Bitplane Scientific (Oxford Instruments company/ USA).

For cell culture experiment: ImageJ 1.53Java1.8.0_331[64-bit] Software (https://imagej.nih.gov/ij/download.html) was used to quantify the mean fluorescence intensity (MFI).

For flow cytometry data analyses FlowJo Software V10.6.1 (BD Life Sciences) was used.

For scRNAseq:
scRNAseq Libraries were sequenced on a HiSeq 4000 Illumina platform. Base calls were converted to fastq reads using the wrapper 'mkfastq' of the software Cell Ranger (10x Genomics, USA; v3.0.2)). Template switch oligo sequence and poly(A) sequence contaminations on Read2 were trimmed with cutadapt (v2.3). zUMIs pipeline (v2.0) was used for subsequent processing of reads. Read2 sequences were mapped to the human genome reference GRCh38 release 91 from ensemble using STAR (v2.6.0c).

Data analysis

For scRNAseq:
Data analysis was performed with Seurat 3.0. To delineated cell barcodes representative of quality cells from barcodes of apoptotic cells or

background RNA, the following thresholds had to be passed: having at least 17.5% of transcripts arising from intronic, i.e. unspliced reads, indicative of nascent mRNA; profiling of more than 3000 UMIs per cell barcode; and less than 20% of their transcriptome being of mitochondrial origin. Louvain cluster analysis was performed to identify cell types using the R package Seurat (version 3). Multiplet cell populations, identified as having a transcriptomic gene expression profile that resembled the resulting combination of 2 disparate cell type signatures that already existed in the dataset, and low UMI cell populations were not included in downstream analyses. A shared nearest neighbor network was created based on Euclidean distances between cells in multidimensional PC space and a fixed number of neighbors per cell, which was used to generate a 2-dimensional Uniform Manifold Approximation and Projection UMAP for visualization. Gene expression between groups of cells was compared using a Wilcoxon Rank Sum test with Bonferroni correction of p-values for multiple testing.

For other statistical analysis:
For not normally distributed data, Mann Whitney was used for unpaired and Wilcoxon signed rank for paired comparison. When multiple groups were compared, we tested first for normality and then used ANOVA with Tukey correction for multiple testing for unpaired and repeated measures ANOVA for paired data. The exact test used can be found in the respective figure legend. Statistical analysis of the single cell data set incl. the software used is described in the methods section, as it is part of the workflow. Throughout, an (adjusted) p-value less than 0.05 was considered significant. Statistical analysis was performed with GraphPad Prism 9.3.14718.0 (GraphPad Software Inc). Comparison between groups was done as indicated.

For manuscripts utilizing custom algorithms or software that are central to the research but not yet described in published literature, software must be made available to editors and reviewers. We strongly encourage code deposition in a community repository (e.g. GitHub). See the Nature Research guidelines for submitting code & software for further information.

Data

Policy information about availability of data

All manuscripts must include a data availability statement. This statement should provide the following information, where applicable:
- Accession codes, unique identifiers, or web links for publicly available datasets
- A list of figures that have associated raw data
- A description of any restrictions on data availability

Sequencing data was deposited to the GEO repository under the accession number GSE141939 and are publicily available.
Sequencing reads were mapped to the human genome reference GRCh38 release 91 from ensemble.

Field-specific reporting

Please select the one below that is the best fit for your research. If you are not sure, read the appropriate sections before making your selection.

☒ Life sciences ☐ Behavioural & social sciences ☐ Ecological, evolutionary & environmental sciences

For a reference copy of the document with all sections, see nature.com/documents/nr-reporting-summary-flat.pdf

Life sciences study design

All studies must disclose on these points even when the disclosure is negative.

Sample size

Cell culture experiments:
Bronchial brushes from 68 patients with idiopathic pulmonary fibrosis (IPF), 25 patients with nonUIP fibrotic ILD and 18 healthy volunteers of an older age (>50 years) were obtained for cell culture experiments. Outgrowth of airway basal cells was used for lentiviral transductions, bronchosphere experiments, humanized mouse model and homogenates for Western Blot.

Lung explant tissues of in total 20 patients with IPF, 10 patients with COPD and normal tissue from 5 donor lungs were used for immunohistochemistry, confocal laser microscopy, fibroblast isolation, and lung homogenates for Western Blot.

For the single cell RNA sequencing experiment, sample size calculation was not performed; a comparable sample size to scRNAseq publications of the lung fibrosis field (Morse et al. ERJ 2019: 3 IPF vs 3 Control; Reyfman et al. AJRCCM 2019: 8 CTRL vs 4 IPF+4non-IPF ILD) was chosen. Therefore, scRNAseq was performed on bronchial epithelial cells derived from nine patients with IPF and six patients with nonUIP fibrotic ILDs.

We report findings of a newly established mouse model based on intratracheal injection of human cells. Our initial power calculations used animal numbers (11 mice per arm) which our institute has established for compound testing in the bleomycin induced pulmonary fibrosis. These calculations served well for compound testing in our new model. For testing the effect of different cell types less numbers are needed.For
the establishment of the humanized mouse model for IPF we used Rag2-/- mice with (BL6 background) with the following groups: bleomycin + IPF-ABCs (n=11) compared to mice challenged with bleomycin + HV-ABCs (n=11) or bleomycin alone (n=21) or controls (n=6). Ashcroft Score and hydroxyproline level were analysed in 2 replicates. To track engraftment of human ABCs bioluminescence measurements were performed. Mice (n=10, 3 replicates) were challenged with human IPF-ABCs transfected with a luciferase and GFP encoding vector. To test whether human IPF-ABCs promote bleomycin induced fibrosis also in NRG mice, n=11 NRG mice per group were used (control, bleomycin, bleomycin + IPF ABCs, 2 replicates). Pulmonary fibrosis was quantified by Ashcroft Score and hydroxyproline level. We also used our xenograft mouse model to test the efficacy of the SRC-inhibitor saracatinib in vivo. NRG mice were challenged with bleomycin and IPF-ABCs and treated either with saracatinib or vehicle. Treatment with saracatinib was started either at day 4 or day 8. For this experiment n=11 NRG mice per arm

were used (2 replicates). To test the effect of src expression on pulmonary fibrosis in vivo, we transduced IPF-ABCs to either overexpress, or knock-down c-src expression. We used n=6 NRG mice per arm (3 groups OE, WT, KD) and 2 replicates.

We did not perform a sample size calculation for the studies using our organoid model. We used primary cells directly derived from patients. Because of that we observed a substantial variability in their response. We corrected for these variabilities by using high numbers of patients from whom the cells were recruited. To test the profibrotic effect of human IPF-ABCs and their crosstalk with human fibroblasts lower sample sizes (n=5 different cell lines with 3 technical repeats) were sufficient since we applied paired comparisons. Similarly, for compound testing also lower sample sizes (n=5) are needed to demonstrate statistical significance.

Data exclusions	For scRNAseq:<br>Cell barcodes representative of quality cells from barcodes of apoptotic cells or background RNA were excluded. The following thresholds had to be passed: having at least 17.5% of transcripts arising from intronic, i.e. unspliced reads, indicative of nascent mRNA; profiling of more than 3000 UMIs per cell barcode; and less than 20% of their transcriptome being of mitochondrial origin.
Replication	No additional experiments other than the experiments reported here, were performed. Replication numbers are given in the manuscript and we report also on their success.
Randomization	Mice were randomly distributed<br><br>For scRNAseq: Patients were allocated in two major experimental condtions, IPF and nonUIP fibrotic ILDs as disease controls. Patients are representative of the two populations of interest. Patients were recruited based on availability.<br><br>For the experiments in which we tested the effect of cells in our in vitro or in vivo models all samples that we were able to obtain were allocated to the experiments. For later experiments in which we tested the therapeutic effects of c-src modulation or of compounds we used a different approach. All primary cell lines (IPF-ABCs) which were generated by our lab were routinely tested for their capacity to generate bronchospheres in our organoid model. For in vitro experiments in which we tested therapeutic effects of compounds or c-src modulation, we used IPF-ABC lines which produced moderate to high numbers of organoids. For the in vivo experiments in which we tested the effect of saracatinib in our mouse model we used IPF-ABC lines which generated high organoid counts.
Blinding	Data of murine experiments were analyzed by a blinded pathologist. The investigators were blinded in regards to the different cell types which they intratracheally injected. In the experiments in which we tested the effect of the medical compound saractinib in our mouse model blinding of the investigators administering the compounds was not possible because we could not obtain blinded medication.

Reporting for specific materials, systems and methods

We require information from authors about some types of materials, experimental systems and methods used in many studies. Here, indicate whether each material, system or method listed is relevant to your study. If you are not sure if a list item applies to your research, read the appropriate section before selecting a response.

Materials & experimental systems

n/a	Involved in the study	
☐	☒	Antibodies
☐	☒	Eukaryotic cell lines
☒	☐	Palaeontology and archaeology
☐	☒	Animals and other organisms
☐	☒	Human research participants
☒	☐	Clinical data
☒	☐	Dual use research of concern

Methods

n/a	Involved in the study	
☒	☐	ChIP-seq
☒	☐	Flow cytometry
☒	☐	MRI-based neuroimaging

Antibodies

Antibodies used	Immunohistochemistry: C-SRC (Abcam, Cat#ab47405, Lot#GR3232598-2), CK5/6 (Dako Omnis, Cat#GA78061-2), P63 (Calbiochem, #PC373), anti-human anti-αvβ6 (kindly provided by Shelia Violette, Biogen Idec), Negative Control Mouse IgG1 antibody (Dako Omnis, Cat#GA75066-2, Lot#10107400), FLEX Universal Negative Control Rabbit Dako Omnis, Cat#IS60061-2), FLEX Universal Negative Control, Mouse (Dako Omnis, Cat# GA75066-2, Lot#10107400), Goat IgG anti-Mouse IgG (H+L)-Biotin (Dianova, Cat#115-065-003, Lot#), Goat IgG anti-Rabbit IgG (H+L)-Biotin (Dianova, #111-065-003)<br><br>Immunofluorescence: SRC (Cell Signaling, Cat#2109S, Lot#7, Clone#36D10), CK5/6 (Dako Omnis, Cat#GA78061-2), CK8 (Abcam, Cat# ab53280, Lot# GR3241174-3, Clone# EP1628Y), AQP5 (Santa-Cruz, Cat#sc-9891, Lot#F1015, Clone#C-19), eGFP (CCell singaling, Cat#2956, Lot#4, Clone# D5.1), Anti-Alpha smooth muscle Actin (Abcam, Cat#ab124964, Lot# GR181740-64, Clone# EPR5368), Vimentin (Merck, Cat#V4630, Lot#069M4800V), Rabbit IgG, polyclonal – Isotype Control (Abcam, Cat#37415, Lot# GR3257876-2),

FLEX Universal Negative Control, Mouse (Dako Omnis, Cat# GA75066-2, Lot#10107400), Goat IgG Isotype Control (Thermo Fisher Scientific, Cat#31245), CK5 (Abcam, Cat#193895, Lot# GR3254308-5, Clone# EP1601Y), CK6 (Abcam, Cat#ab93279, Lot# GR237556-13, Clone# EPR1602Y), MUC5AC (Bioss Antibodies, Cat#bs-7166R-Cy3, Lot# AF01258750), Acetyl-α-Tubulin (Lys40) (Cell Signaling, Cat#12152, Lot#1, Clone#6-11-B-1), CK17(NSJ Bioreagents, Cat# V2176-100UG, Lot# V2176SAF-191210, Clone#E3), Donkey IgG anti-Rabbit IgG (H+L)-Cy5 (Jackson Immuno Research, Cat#711-175-152, Lot#143470), Donkey Fab anti-Mouse IgG (H+L)-Alexa Fluor 488 (Jackson Immuno Research, Cat#715-547-003, Lot#132336), Donkey IgG anti-Rabbit IgG (H+L)-Cy3 (Jackson Immuno Research, Cat#711-165-152, Lot#136182), Donkey IgG anti-Mouse IgG (H+L)-Cy5 (Jackson Immuno Research, Cat#715-175-150, Lot#142571), Donkey IgG anti-Goat IgG (H+L)-Cy3 (Jackson Immuno Research, Cat#705-165-147, Lot#135077), Donkey Fab anti-Rabbit IgG (H+L)-Alexa Fluor 488 (Jackson Immuno Research, Cat#711-547-003, Lot#156827)

Western Blot: C-SRC (Abcam, Cat#ab47405, Lot#GR3232598-2), Phospho-Src (Cell Singaling, Cat#2105S, Lot#9), Type I Collagen (Southern Biotec, Cat#SAB-1310-01, Lot#B2918V688), α-SMA (Merck Millipore, Cat#CBL171, Lot#2718341, Clone#ASM-1), Fibronectin (Enzo Life Sciences, Cat#BML-FG6010, Lot#12021411, Clone#DH1), phospho-p44/42 (Thr202/Tyr204) (Cell Signaling, Cat#9106, Lot#30), total-p44/42 (Cell signaling, Cat#4695, Lot#14), phospho-EGFR (Tyr845) (Zytomed, Cat#205-0235), Phospho-EGFR (Tyr992) (Cell Signaling, Cat#2235, Lot#L9020313), total EGFR (Biozol, Cat#GTX628887, Clone#GT133), GAPDH (Santa Cruz, Cat# sc-32233, Lot# J2020, Clone#6C5), α –Tubulin (Cell Signaling, Cat#2125, Lot#11, Clone#11H10), goat anti rabbit (H+L)-HRP conjugate (BioRad, Cat#1706515, Lot#006328A), goat anti mouse (H+L)-HRP conjugate (BioRad, Cat#1706516, Lot#006326A)

Flow Cytometry: EpCAM (Miltenyi, Cat#30-11-000, Lot#5201108357, clone# REA764), CK5 (abcam, Cat#ab224985, Lot# GR3403305-1, Clone# EP1601Y), CK17 (Abcam, Cat# ab185032, Lot# GR174675-3, Clone# EP1623), Prom1 (Miltenyi, Cat#130-113-668, Lot#5220306939, Clone# AC133)

Validation

The specifity of primary antibodies in IHC, FC and IF were tested by staining against IgG controls.

Immunohistochemistry:
C-SRC (Abcam, Cat#ab47405). Certificate of compliance is available. References: Chen JX et al. TRIM47 promotes malignant progression of renal cell carcinoma by degrading P53 through ubiquitination. Cancer Cell Int 21:129 (2021); Ebeid DE et al. PIM1 Promotes Survival of Cardiomyocytes by Upregulating c-Kit Protein Expression. Cells 9:N/A (2020).
CK5/6 (Dako Omnis, Cat#GA78061-2). Validated by the manufacturer
P63 (Calbiochem, #PC373). Validated by manufacturer. References: Xu B, Wang L, Borsu L, Ghossein R, Katabi N, Ganly I, Dogan S. A proportion of primary squamous cell carcinomas of the parotid gland harbour high-risk human papillomavirus. Histopathology. 2016 Dec;69(6):921-929. doi: 10.1111/his.13027. Epub 2016 Aug 23. PMID: 27374168; PMCID: PMC5115934; Xu B, Aneja A, Ghossein R, Katabi N. Predictors of Outcome in the Phenotypic Spectrum of Polymorphous Low-grade Adenocarcinoma (PLGA) and Cribriform Adenocarcinoma of Salivary Gland (CASG): A Retrospective Study of 69 Patients. Am J Surg Pathol. 2016 Nov;40(11):1526-1537. doi: 10.1097/PAS.0000000000000705. PMID: 27454943; PMCID: PMC5069130.
anti-human anti-αvβ6 (kindly provided by Shelia Violette, Biogen Idec). References: Shea BS, Probst CK, Brazee PL, Rotile NJ, Blasi F, Weinreb PH, Black KE, Sosnovik DE, Van Cott EM, Violette SM, Caravan P, Tager AM. Uncoupling of the profibrotic and hemostatic effects of thrombin in lung fibrosis. JCI Insight. 2017 May 4;2(9):e86608. doi: 10.1172/jci.insight.86608. PMID: 28469072; PMCID: PMC5414562; Puthawala K, Hadjiangelis N, Jacoby SC, Bayongan E, Zhao Z, Yang Z, Devitt ML, Horan GS, Weinreb PH, Lukashev ME, Violette SM, Grant KS, Colarossi C, Formenti SC, Munger JS. Inhibition of

Eukaryotic cell lines

Policy information about cell lines

Cell line source(s)

The A549 cell line was purchased from Merck, #86012804-1VL

Authentication

Cell line were not authenicated.

Mycoplasma contamination

Cells were not tested for mycoplasma contamination.

Commonly misidentified lines
(See ICLAC register)

No commonly misidentified cell lines were used in the study.

Animals and other organisms

Policy information about studies involving animals; ARRIVE guidelines recommended for reporting animal research

Laboratory animals

B6;129Sv-Rag2tm1Fwa/ZTM (Rag2-/-), NOD.Cg-Rag1tm1Mom Il2rgtm1Wjl/SzJZtm (NRG), NOD.Cg-Prkdcscid Il2rgtm1Wjl Tg(CAGGS-VENUS)1/Ztm (Venus-NSG). RAG2-/- mice used in this study were maintained on the C57BL/6J background for >6 generations. All RAG2-/-, NRG and Venus-NSG mice used in this study were male and 8-12 weeks old. Mice were housed under standardized conditions (room temperature 22 + 2 °C, relative humidity 55 + 15 %) on 12 hours light-dark cycle with access to food and water ad libitum

Wild animals

No wild animals were used in the study.

Field-collected samples	No field collected samples were used in the study.
Ethics oversight	All mouse procedures were conducted in accordance in with the German law for animal protection and the European Directive 2010/63/EU and were approved by the respective local government (Regierungspräsidium Freiburg, Germany; AZ: 35-9185.81/ G-14/17) and Lower Saxony State Office for Consumer Protection and Food Safety in Oldenburg/Germany (LAVES); AZ: 33.12-42502-04-15/1896 and AZ: 33.19-42502-04-15/2017), AZ:33.19-42502-04-17/2612).

Note that full information on the approval of the study protocol must also be provided in the manuscript.

Human research participants

Policy information about studies involving human research participants

Population characteristics	In total, bronchial brushes from 68 patients with idiopathic pulmonary fibrosis (IPF), 25 patients with nonUIP fibrotic ILD and 18 healthy volunteers of an older age (>50 years) were obtained. Only patients with an idiopathic UIP and HRCT consistent with a "definite" UIP pattern were included. The covariate-relevant population characteristics of the human research participants can be found in supplemental table. We did not notice any age or sex-dependent effects. In addition, lung explant tissues of in total 20 patients with IPF, 10 patients with COPD and normal tissue from 5 donor lungs were used for fibroblast isolation, lung homogenates and immunohistochemistry. IPF diagnosis and other ILD diagnosis was established by a multidisciplinary board according to the American Thoracic Society/ European Respiratory Society criteria and was later determined to be consistent with recent guidelines
Recruitment	Patients were recruited at the ILD clinics of Hannover Medical School (approved IRB protocols #2518-2014 and #2923-2015) and University Hospital Freiburg ()approved IRB protocols #239/12 and #03/10), both are tertiary referral centres. Patients underwent standardized bronchoscopy during routine diagnostic work-up. Explanted lung tissue were obtained from donors with end stage disease undergoing lung transplantation.
Ethics oversight	All experiments with human tissue samples were performed under protocols approved by the Institutional Review Boards at Hannover Medical School and University Medical Center Freiburg. All patients and healthy volunteers signed informed consent prior to inclusion to the study.

Note that full information on the approval of the study protocol must also be provided in the manuscript.
